# Editorial: Isolated otolith dysfunction and vertigo

**DOI:** 10.3389/fneur.2022.1030513

**Published:** 2022-10-26

**Authors:** Toshihisa Murofushi, Myung-Whan Suh, Leonardo Manzari

**Affiliations:** ^1^Department of Otolaryngology, Mizonokuchi Hospital, Teikyo University School of Medicine, Kawasaki, Japan; ^2^Department of Otolaryngology-Head and Neck Surgery, Seoul National University Hospital, Seoul, South Korea; ^3^MSA ENT Academic Center, Cassino, Italy

**Keywords:** otolith, VEMP, saccule, utricle, semicircular canal, vHIT

## Introduction

The otolith organs, saccule, and utricle sense linear acceleration, while the semicircular canals, lateral, anterior, and posterior semicircular canal sense angular acceleration (1–3). It is well known that acute unilateral semicircular canal dysfunction causes a spinning vertigo attack and can be detected using clinical tests such as caloric tests and rotatory tests (4). Neurophysiological study of eye movements by electrical stimulation of each canal afferent has confirmed the relationship between semicircular canal dysfunction and spontaneous nystagmus (5). On the other hand, symptoms and signs due to otolith dysfunction have not been clearly defined although non-spinning vertigo, translation, tilt, floating, or flipping-over sensation has been considered to be caused by otolith disorders (6–9). Clinical tests for assessment of otolith function at clinics were not available until 1992 (10). Therefore, “otolithic vertigo” was not widely accepted although some investigators have proposed “otolithic vertigo” as a clinical entity (2, 3, 6, 7, 11). Since 1992 the vestibular evoked myogenic potential (VEMP) has been developed as a clinical test of the otolith organs (3, 10, 12). First, cervical VEMP (cVEMP) has been applied as a test of saccular function, and later ocular VEMP (oVEMP) as a test of utricular function. Another important aspect of the diagnosis of isolated otolith dysfunction and vertigo is to exclude vertical semicircular canal dysfunction, which the development of vHIT (video head-impulse test) has enabled (13, 14). These and other tools that enable the diagnosis of “otolithic vertigo” or “vertigo/dizziness due to isolated otolith dysfunction” as a clinical entity are now ready for development. This Research Topic invited investigators and clinicians to submit “*Isolated otolith dysfunction and vertigo*”. The collection includes four original papers that discuss issues toward the establishment of this clinical entity.

## Summary of articles

Waissbluth and Oyarzún reported a case that was considered to have “isolated unilateral saccular dysfunction”. A 20-year-old woman presented with frequent episodic dizziness. She showed unilateral absence of cVEMP with normal oVEMP and normal vHIT. This report outlines some important points for the diagnosis of “vertigo/dizziness due to isolated otolith dysfunction”. As this patient was young and showed a unilateral absence of cVEMP, it was convincing that her findings were pathological. However, one should also note that appropriate cVEMP recording requires adequate SCM (sternocleidomastoid muscle) contraction without left-right difference and preferably, normalization of amplitudes according to muscle activation levels (12). Further young cases outlining unilateral abnormal findings of cVEMP and/or oVEMP that have been obtained with appropriate methods and objective findings and that discuss non-spinning vertigo attacks as subjective symptoms are required.

Kim et al. report on a valuable cross-sectional study concerning the involvement patterns of vestibular end-organs in vestibular patients. Their reports showed that vestibular patients could show otolith dysfunction with preserved canal function. At the same time, this report suggested that abnormal test results of otolith function (cVEMP and/or oVEMP) with normal test results of canal function (vHIT and caloric test) should not be enough for diagnosis of “vertigo/dizziness due to isolated otolith dysfunction”. Especially for the diagnosis of idiopathic vertigo/dizziness due to isolated otolith dysfunction, thorough exclusion of various known vestibular diseases and spinning vertigo episodes is required.

Murofushi et al. report on isolated otolith dysfunction in patients with persistent postural-perceptual dizziness (PPPD). The majority of PPPD patients in this study had otolith dysfunction and many of them included isolated otolith dysfunction. Idiopathic vertigo/dizziness due to isolated otolith dysfunction could be a precipitating condition of PPPD. This study suggested that the association of PPPD with isolated otolith dysfunction should be further clarified. However, this study included patients older than 65 years of age. These patients should be carefully diagnosed.

Xu et al. reported vestibular disorders in patients with obstructive sleep apnea syndrome (OSAS). Patients with OSAS had much higher abnormality rates in otolith tests (cVEMP and oVEMP) than canal tests (caloric test and vHIT). Although their study did not directly mention isolated otolith dysfunction, it suggested that the otolith organ might be more vulnerable than the semicircular canal to hypoxic events. One should be careful with the placement of electrodes in OSAS patients because their obesity might make proper placement difficult.

## Issues to be solved for the establishment of a new clinical entity “idiopathic vertigo/dizziness due to isolated otolith dysfunction”

What is required to establish a new clinical entity “Idiopathic vertigo/dizziness due to isolated otolith dysfunction (IOV/D)”? As Suh and Murofushi propose, both subjective symptoms such as non-spinning, translation, tilt, floating, or flipping-over sensation without spinning vertigo attacks, and objective test findings such as abnormal cVEMP and/or oVEMP with normal vHIT, will be required (15). While subjective visual vertical/horizontal test (SVV/H) might be also considered in the diagnostic criteria, one should note that SVV/H can be affected by central nervous system lesions such as the thalamus and parieto-insular vestibular cortex (16).

The credibility of VEMP test results is an essential aspect of developing this field. To exclude false positivity, at the current stage, only patients younger than 70 (or 65) years of age with unilateral abnormal findings should be enrolled as IOV/D, because VEMP responses could be affected by aging and bilateral abnormal findings might be due to non-vestibular factors (17). Proper test procedures must be applied and, at the same time, a thorough differential diagnosis of other vestibular diseases such as Meniere's disease must be undertaken. Large-sized surveys of IOV/D under the provisional diagnostic criteria need to be undertaken in the future.

## Author contributions

TM wrote the manuscript. M-WS and LM edited the manuscript. All authors contributed to the article and approved the submitted version.

## Conflict of interest

The authors declare that the research was conducted in the absence of any commercial or financial relationships that could be construed as a potential conflict of interest.

## Publisher's note

All claims expressed in this article are solely those of the authors and do not necessarily represent those of their affiliated organizations, or those of the publisher, the editors and the reviewers. Any product that may be evaluated in this article, or claim that may be made by its manufacturer, is not guaranteed or endorsed by the publisher.
